# Prevalence of tuberculosis and associated risk factors in the Central Prison of Mbuji-Mayi, Democratic Republic of Congo

**DOI:** 10.1186/s41182-016-0030-9

**Published:** 2016-09-20

**Authors:** Guillaume MuasaPatoka Kalonji, Gérard De Connick, Léon Okenge Ngongo, Dieudonné Kazumba Nsaka, Thierry Kabengele, Félicien Tshimungu Kandolo, Félicien Ilunga-Ilunga, Albert Adelin, Didier Giet

**Affiliations:** 1 Department of Public Health Sciences, University of Liege, Sart Tilman (B23), 4000 Liege, Belgium; 2 Liege Criminology School Jean Constant, University of Liege, Liege, Belgium; 3 Lodja University of Sciences and Technologies, Lodja, Democratic Republic of Congo; 4 Mbuji-Mayi Higher Institute of Medical Engineering, Mbuji-Mayi, Democratic Republic of Congo; 5 Medical Centre of Mbuji-Mayi Central Prison, Mbuji-Mayi, Democratic Republic of Congo; 6 Kinshasa Higher Institute of Medical Engineering, Kinshasa, Democratic Republic of Congo; 7 Department of General Practice, University of Liege, Liege, Belgium

**Keywords:** Prevalence, Tuberculosis, Prison, Democratic Republic of Congo

## Abstract

**Background:**

Tuberculosis still remains a major public health concern in several provinces of the Democratic Republic of Congo, especially in prison settings. The present study aimed at determining tuberculosis (TB) prevalence and associated risk factors in inmates of the Mbuji-Mayi Central Prison.

**Methods:**

This cross-sectional study was performed over a 6-month period (January to June 2015) in Mbuji-Mayi Central Prison. A total of 733 inmates were screened systematically for TB. The diagnosis was based on clinical examination and bacteriological tests.

**Results:**

Tuberculosis was diagnosed in 130 inmates, what amounts to a 17.7 % prevalence (95 % confidence interval [CI] 15.1–20.6 %). The mean age ± SD of infected inmates was 31 ± 9.5 years old, and 94.8 % of them were male. Inmates were detained for a median period of 24 months (range: 3 months to 12 years). A cough lasting more than 2 weeks, body temperature higher than 39 °C, and weight loss were the predominating clinical signs. Factors independently associated with TB infection were overcrowding; highest population attributable fraction ([PAF] 88.2 %; adjusted odds ratio [OR] 9.8 [95 % CI 3.1–31.6]); malnutrition (body mass index of less than 18.5 kg/m^2^) (PAF 35.6 %; adjusted OR 2.1 [1.3–3.0]); and a detention period equal to or greater than 12 months (PAF 38.7 %; adjusted OR 2.1 [1.4–3.1]).

**Conclusions:**

Improving detention and sanitary conditions, as well as providing an adequate and early healthcare, are urgently needed to reduce TB prevalence in the prison environment.

## Background

Despite being on the wane over the past few years, tuberculosis (TB) remains a lethal transmissible disease around the world [1]. According to the World Health Organization (WHO), 9.6 million people were infected by TB in 2014 and 1.5 million died of the disease [2]. In Africa, the situation has become alarming in the last few years due to the poor socioeconomic context and to the incidence of human immunodeficiency virus (HIV) infections. At least 80 % of TB patients live in sub-Saharan Africa and in Asia. More than 95 % of deaths caused by TB occur in low- and middle-income countries. Tuberculosis is taking on alarming proportions in the prison environment, as poor conditions of detention facilitate its transmission. Indeed, the risk of TB infection would be 100 times greater in prison than in communities [3, 4]. Prison environment is often characterized by overcrowding, nutritional deficiencies, and inadequate healthcare; these factors turn prison into a perfect breeding ground for TB dissemination [5]. In Africa, TB is endemic, especially in prison settings [6–8], and the Democratic Republic of Congo (DRC) is one of the countries most affected. According to the WHO, the annual incidence reached 325 cases per 100,000 population (116,894 new TB cases) with a prevalence of 532 cases for every 100,000 inhabitants in 2014; the case-fatality rate was estimated at 69 per 100,000 population [2]. In DRC, TB control is coordinated by the National TB Control Programme. In 2015, the Kasaï-Oriental Coordination registered 13,341 TB cases, all clinical forms included, in Mbuji-Mayi [9]. The first national survey on resistance to anti-TB drugs was implemented in July 2015; its preliminary results identified Mbuji-Mayi as being the region the most affected by resistant TB, with a prevalence of 15 and 37 % in new cases and former patients, respectively [9]. Prevalence of resistance is three times and five times above the national average in, respectively, former and newly diagnosed patients [9]. Prison settings have been often identified as important but neglected reservoirs for TB [10]. Most studies conducted on TB in Africa prisons showed higher prevalence of smear-positive pulmonary TB in prisons compared to in the civilian population [11–14]. The present study aimed at determining the prevalence of TB and associated risk factors in the Mbuji-Mayi Central Prison. To our knowledge, no study has ever investigated these parameters in the country to date. This work highlights the necessity of screening any inmate upon admission and of improving conditions of detention, in order to significantly reduce TB prevalence; indeed, preventive measures and treatment may be implemented.

## Methods

### Study setting

The study was performed in the Mbuji-Mayi Central Prison, municipality of Diulu, one of the five urban units of Mbuji-Mayi (former Kasaï Oriental province, now divided into three administrative regions), DRC. The prison was built in 1952 and was originally designed to accommodate 150 inmates, but more than 800 prisoners were jailed in 2014 [15]. These old buildings consist of eight dorms (no light nor ventilation in them), each housing around 70 inmates, and several administrative offices.

### Population and type of study

This cross-sectional study included 733 inmates, systematically screened for TB over a 6-month period (from January 1 until June 30, 2015).

### Inclusion criteria

All prison inmates were involved in the study, including TB patients under treatment before screening. Any clinical suspicion of TB was confirmed by bacteriology (positive microscopy), and anti-TB therapy was immediately started.

### Data collection

Data were collected by health professionals assigned to the prison medical center; they were previously informed on the terms of the survey and organizational measures, as well as on personal protective equipment, mandatory to prevent any contamination (respiratory mask, gloves, and overalls). Investigators were required to be up to date with vaccination. Every prisoner was examined and further questioned by investigators: general, sociodemographic, clinical, and epidemiological data were collected through a structured questionnaire (pretested before starting the investigation). The diagnosis was based on clinical and para-clinical data. The prison chief medical officer was in charge of clinical examinations. Para-clinical tests (pulmonary X-ray, Ziehl-Nielsen technique, and auramine dark-field microscopy performed on sputum) were performed at the provincial laboratory of the National TB Control Programme. The following characteristics were taken into account: age, sex, height (meters), weight (kilograms), marital status, level of education, inmate’s status, length of detention, clinical signs (weight loss, anorexia, asthenia, duration of cough, fever, thoracic pain, night sweats, sputum, and hemoptysis) and conditions of detention (number of detainees per dorm). A dorm was considered as overcrowded if more than 50 prisoners were housed in a 40 m^2^-cell. The body mass index (BMI) characterized inmates’ nutritional status: malnutrition was assessed by a BMI less than 18.5 kg/m^2^.

### Statistical analyses

Descriptive statistics were first used to analyze the data. Either a chi-square test or a Fisher exact test was performed on contingency tables. A univariate analysis allowed estimating crude odds ratios (OR) and their 95 % confidence intervals (95 % CI). Factors independently associated with TB occurrence were then investigated through a stepwise logistic regression analysis. Variables included in the model were selected via a stepwise procedure based on the likelihood ratios; non-significant independent variables (*p* value greater than 0.05) were deleted from the regression model. For each associated factor, the contribution to the disease *population attributable fraction* (PAF) was estimated after adjustment. We calculated the PAF using Levin’s formula: PAF = *P*_e_ (OR_e_ − 1)/[1 + *P*_e_ (OR_e_ − 1)]. *P*_e_ is the prevalence of the exposure, and OR_e_ is the odds ratio of TB due to that exposure. Results were considered as significant for a *p* value less than 0.05. Statistical analyses were performed with STATA 12.0 software.

## Results

### Sociodemographic and clinical features of inmates

Of the 733 prisoners interviewed, 94.8 % were male and their mean age ± SD was 31 ± 9.5 years old. The median length of incarceration was 24 months (IQR 18–52 months). Malnutrition (BMI less than 18.5 kg/m^2^) was noticed in half the inmates. Predominating clinical signs were weight loss and asthenia, as reported in 85.3 and 61.4 % of prisoners, respectively (Table 1).

**Table 1:** Sociodemographic and clinical characteristics of prisoners (*N* = 733)

Parameters	Number	Median (IQR)	Mean ± SD	Percent
Age (years)			31 ± 9.5	
< 30	409			55.8
Sex				
Male	695			94.8
Length of incarceration		24 (18–52)		
< 12 months	420			57.3
BMI (kg/m^2^)				
< 18.5	368			50.2
Overcrowding (>50 prisoners/40 m^2^)				
Yes	620			84.6
Clinical signs				
Cough > 2 weeks	125			17.1
Weight loss	625			85.3
Temperature > 39 °C	115			15.7
Night sweat	109			14.9
Asthenia	450			61.4
Thoracic pain	110			15.0

*SD* standard deviation, *IQR* interquartile range, *BMI* body mass index

### Prevalence of tuberculosis and associated factors—univariate analysis

A diagnosis of TB was made in 130 inmates (23 former and 107 new cases), which means a 17.7 % prevalence (95 % CI 15.1–20.6 %). No significant difference was observed between young (under 30 years of age; 15.7 %) and old (over 30 years of age; 20.4 %) age groups. Tuberculosis particularly affected men, rather than women (18.3 vs. 7.8 %, respectively), although no significant difference was highlighted. The following parameters were considered as risk factors for developing TB, as shown by the univariate analysis (crude ORs) in Table 2: malnutrition (BMI less than 18.5 kg/m^2^ [OR 1.8; 95 % CI = 1.2–2.7]), a length of detention equal to or greater than 12 months (OR 1.9; 95 % CI [1.3–2.7]), and overcrowding (OR 9.4; 95 % CI [2.9–30.2]).

**Table 2:** Factors associated with TB occurrence (*n* = 130)—univariate analysis

Parameter	TB		Crude OR (95 % CI)	*p* value
	*N*	%		
Age (years)				
< 30	64	15.7	1	
≥ 30	66	20.4	1.4 (0.9–2.0)	0.097
Sex				
Male	127	18.3	2.6 (0.7–8.6)	0.116
Female	3	7.8	1	
Length of incarceration (months)				
< 12	58	13.8	1	
≥ 12	72	23.0	1.9 (1.3–2.7)	0.001
BMI (kg/m^2^)				
< 18.5	81	22.0	1.8 (1.2–2.7)	0.003
≥ 18.5	49	13.4	1	
Overcrowding (>50 prisoners/40 m^2^)				
Yes	127	20.5	9.4 (2.9–30.2)	<0.001
No	3	2.7	1	

*TB* tuberculosis, *OR* odds ratio, *CI* confidence interval, *BMI* body mass index

After performing the logistic regression model (multivariate analysis), TB remained statistically associated with the following parameters (Table 3): malnutrition, a length of detention equal to or greater than 12 months, and overcrowding (more than 50 prisoners/40 m^2^). Factors attributable to the risk of TB in the population (PAF) were estimated for these three risk factors. Overcrowding showed the highest PAF (88.2 %), followed by malnutrition (35.6 %) and a length of detention equal to or greater than 12 months (38.7 %), as shown in Table 3.

**Table 3:** Factors associated with TB occurrence (*n* = 130)—multivariate analysis

Parameter	Adjusted OR (95 % CI)	*p* value	PAF (%)
Incarceration period (months)			
< 12	1		
≥ 12	2.1 (1.4–3.1)	0.001	38.7
BMI (kg/m^2^)			
< 18.5	2.1 (1.3–3.0)	0.003	35.6
≥ 18.5	1		
Overcrowding (>50 prisoners/40 m^2^)			
Yes	9.8 (3.1–31.6)	<0.001	88.2
No	1		

*PAF* population attributable fraction

Hosmer-Lemeshow (HL) test = 1.58; *p* = 0.063

## Discussion

Our study aimed at estimating TB prevalence (confirmed by positive microscopy) in the population of Mbuji-Mayi Central Prison and investigating the possible association between sociodemographic characteristics of prisoners, conditions of detention, and occurrence of TB.

The sample included 733 prisoners, mostly men. Tuberculosis prevalence (17.7 %) was elevated compared to other prisons in Africa. Studies in Cameroon (1.2 %), in Ivory coast (5.8 %), in and Ethiopia (10.4 %) showed lower prevalence of smear-positive pulmonary TB in prisons compared to our study [3, 8, 16]. The prevalence in the Mbuji-Mayi Central Prison (130/733 = 17.7 %) is about 33 times higher than the national average (532/100,000 = 0.532 %). Our study confirms the conclusions of a previous Ethiopian survey conducted in 2011, where TB prevalence in prison was sevenfold higher compared to the general community [14]. In Brazil, data collected in Rio state prisons revealed an annual TB prevalence 31 times higher than in the general population, despite the differences between prisons [17]. Other investigations reported a TB prevalence above 20 % in some Malawi prisons [18]. Of 296 Cameroonian individuals tested, 11.5 % were positive for acid-fast bacilli [3]. Combined to inadequate care, especially in a prison environment, TB prevalence remains elevated in DRC.

Sociodemographic characteristics of TB patients were similar to those described in previous studies [17, 19]. Even if no statistical difference was highlighted for age groups and sex in our study, Lilanganee Telisinghe and collaborators observed that TB especially affected young adults, aged between 27 and 37 years old, in South Africa [20]. Similar trends were observed in Pakistan and Malawi [21, 22]. The very low sample size of some categories could likely explain such observation. According to the WHO, the male/female ratio is not relevant for the disease, as women are often outnumbered by men in the prison population [23].

The univariate analysis showed that a length of detention equal to or greater than 12 months is a crucial risk factor to become infected. Overcrowding was also identified as a risk factor, and most prisoners were malnourished, as confirmed by a BMI lower than 18.5 kg/m^2^. A poor diet increases the risk of contracting TB, as body defenses are generally lowered, which explains a higher prevalence of the disease in prison. The same risk factors were identified in Cameroon, Pakistan, and Zimbabwe [3, 21, 24] An incarceration in overcrowded cells, a BMI lower than 18.5 kg/m^2^, and a prior anti-TB therapy were also identified as risk factors for TB in prison [16, 19]. Furthermore, Harris and collaborators [25] concluded that transmission of TB was exacerbated by several factors in the prison environment: a poor diet, overcrowding (generating a poor ventilation), HIV, and other ordinary conditions facilitating the evolution of a latent TB infection into a clinical disease. These authors incriminate a poor financial support and the lack of healthcare organization as factors increasing the probability of a late diagnosis, incomplete therapy, and facilitated transmission. The multivariate analysis confirmed the observations of the univariate approach. Three factors were highlighted as risk factors: malnutrition (low BMI), overcrowding (more than 50 prisoners per 40 m^2^), and a length of detention equal to or greater than 12 months, in accordance with previous reports [26–28]. These factors showed a very high PAF; their suppression would prevent 35.6 to 88.2 % of TB cases recorded in prison. In Mbuji-Mayi prison, overcrowding is a real concern. The buildings were initially designed to accommodate 150 inmates; nevertheless, a recent report mentioned six times as many prisoners [9]. Our observations confirm that conditions of detention and promiscuity are a real time-bomb. Overcrowding enhances the propagation of diseases, and TB is transmitted much more easily than HIV, as physical contact is not required. Unclogging the prison would lead to an 84 %-reduction of TB cases and would break the transmission chain between prisoners, staff, and visitors. A discrimination in the prison itself is often observed. Many TB patients come from economically disadvantaged families, who do not have an easy access to healthcare and often live in poor hygienic conditions. Overcrowding was already identified as a risk factor in previous studies, with the highest PAF compared to other factors [16, 27, 29, 30].

The socioeconomic situation of DRC and the low operating budget allocated to prisons, in general, explain the poor quality healthcare. On a daily basis, either humanitarians or families provide food to prisoners, the latter often being extremely poor. Health professionals should be more involved in the control of TB in the prison environment. There is an urgent need for TB prisoners to be followed and correctly treated in order to interrupt the transmission chain, especially since resistance to treatment is progressing (as mentioned in the recent report of the National TB Control Programme). It is imperative to implement measures enabling a rapid identification of clinical TB cases in prisoners. If they are contagious, adequate isolation conditions are required; therapy should be rapidly implemented as well. Infection control guidance must not only be written but also implemented. Tuberculosis control in prisons, and especially in Mbuji-Mayi, remains a priority and should be an integral part of any public health policy aiming at controlling and eradicating the disease. However, basic hygienic conditions are lacking in prison.

### Limitations of the study

The present study investigated the prevalence of TB and associated risk factors in the context of Mbuji-Mayi prison. The relationship between TB and HIV and/or other health problems such as hepatitis were not assessed.

## Conclusions

Tuberculosis remains a major public health concern, exacerbated by the delay and poor quality of diagnosis combined to a deficient healthcare, particularly in the prison environment. Tuberculosis is a serious disadvantage for developing countries, as it particularly affects young adults. In our study, we identified three factors independently associated with TB occurrence: malnutrition, prolonged length of detention, and overcrowding. Improving conditions of detention and providing an adequate and early healthcare would considerably reduce the incidence of TB in prison. Efforts aiming at controlling the disease must include strategies adapted to the prison environment. Furthermore, it should be the target of a national health policy supported by international organizations.

## Abbreviations

BMI: Body mass index
CI: Confidence interval
COE-IU: Inter-University Ethical Committee
DRC: Democratic Republic of Congo
HIV: Human immunodeficiency virus
MBM: Mbuji-Mayi
No: Number
OR: Odds ratio
PAF: Population attributable fraction
TB: Tuberculosis
WHO: World Health Organization
